# Comparative genomics and concerted evolution of β-tubulin paralogs in *Leishmania *spp

**DOI:** 10.1186/1471-2164-7-137

**Published:** 2006-06-06

**Authors:** Andrew P Jackson, Sue Vaughan, Keith Gull

**Affiliations:** 1 Sir William Dunn School of Pathology, University of Oxford, South Parks Road, Oxford. OX1 3RE, UK; 2 Wellcome Trust Sanger Institute, Wellcome Trust Genome Campus, Hinxton, Cambridgeshire. CB10 1SA, UK

## Abstract

**Background:**

Tubulin isotypes and expression patterns are highly regulated in diverse organisms. The genome sequence of the protozoan parasite *Leishmania major *contains three distinct β-tubulin loci. To investigate the diversity of β-tubulin genes, we have compared the published genome sequence to draft genome sequences of two further species *L. infantum *and *L. braziliensis*. Untranscribed regions and coding sequences for each isoform were compared within and between species in relation to the known diversity of β-tubulin transcripts in *Leishmania *spp.

**Results:**

All three β-tubulin loci were present in *L. infantum *and *L. braziliensis*, showing conserved synteny with the *L. major *sequence, hence confirming that these loci are paralogous. Flanking regions suggested that the chromosome 21 locus is an amastigote-specific isoform and more closely related (either structurally or functionally) to the chromosome 33 'array' locus than the chromosome 8 locus. A phylogenetic network of all isoforms indicated that paralogs from *L. braziliensis *and *L. mexicana *were monophyletic, rather than clustering by locus.

**Conclusion:**

*L. braziliensis *and *L. mexicana *sequences appeared more similar to each other than each did to its closest relative in another species; this indicates that these sequences have evolved convergently in each species, perhaps through ectopic gene conversion; a process not yet evident among the more recently derived *L. major *and *L. infantum *isoforms. The distinctive non-coding regions of each β-tubulin locus showed that it is the regulatory regions of these loci that have evolved most during the diversification of these genes in *Leishmania*, while the coding regions have been conserved and concerted. The various loci in *Leishmania *satisfy a need for innovative expression of β-tubulin, rather than elaboration of its structural role.

## Background

*Leishmania *spp. (Trypanosomatidae: Kinetoplastida) are vector-borne parasites of Man worldwide. The precise pathology of leishmaniasis varies across the world depending on which species and strain are concerned, but many result in acute tissue necrosis and can be fatal. The genome sequence of the aetiological agent of old-world cutaneous leishmaniasis, *L. major*, was recently completed [1] and this study addresses the impact of the genome sequence on our understanding of β-tubulin repertoire in *Leishmania*. β-tubulin is one half of the α/β tubulin dimer that comprises eukaryotic microtubules and has been interesting to molecular parasitologists as a model for gene regulation [2], motility [3] and transcription [4] in kinetoplastids and as a potential drug target [5,6].

The *L. major *genome sequence established that there are three β-tubulin loci on chromosomes 33, 21 and 8 respectively, and a single α-tubulin locus on chromosome 13. The genome project corroborated previous investigations that predicted a tandem array of duplicated β-tubulin genes at one locus, and two other singleton genes [7,8], in contrast to the related kinetoplastid parasite, *Trypanosoma brucei*, which has a single β-tubulin locus [9]. These loci were expected based on distinct transcripts in *L. major *that possessed characteristic length and specific expression profiles relating to the life stage of the parasite [2]. Both *L. major *and *L. mexicana *possess an amastigote-specific isoform that is solely expressed in mammalian macrophage [10], and this apparent similarity suggests that all *Leishmania *spp. may have a diverse β-tubulin repertoire. In this study, draft genome sequences for two further species, *L. infantum *and *L. braziliensis*, causes of old-world visceral and new-world muco-cutaneous leishmaniases respectively, were searched for homologs to the three *L. major *loci.

Transcription in kinetoplastids is polycistronic and individual promoter regions for each gene are absent [11,12]. Regulation of gene expression takes place post-transcriptionally through recognition of motifs in the untranscribed regions (UTRs) resulting in differential processing of transcripts and modulation of transcript decay [13-15]. This being so, the genomic environment around coding sequences is of interest when inferring operational differences and β-tubulin isoforms can be expected to differ in their 5' and 3' UTRs if they are differentially expressed. In this study, the flanking regions around β-tubulin loci were compared between and within species to identify relative affinities and unique features.

The presence of three β-tubulin loci in *Leishmania *spp. raises two related questions: why several loci exist in *Leishmania *when these are not necessary in other kinetoplastids and what, if any, are the functional differences between loci. In the event of divergence among these loci in the three *Leishmania *spp., the relative change in coding and untranscribed regions will determine if functional diversification has been derived from structural innovation, as in the case of vertebrate globins or immunoglobulins [16], or whether structurally conserved duplicates are expressed distinctly, either spatially or temporally, as with polyploid cotton [17]. As well as comparing flanking regions to illuminate these issues, primary sequences of all isoforms were analysed to identify the molecular signatures of evolutionary processes, such as recombination, natural selection and gene duplication, which have shaped β-tubulin sequences in *Leishmania*.

## Results

### Comparative genomics

**Figure 1:**
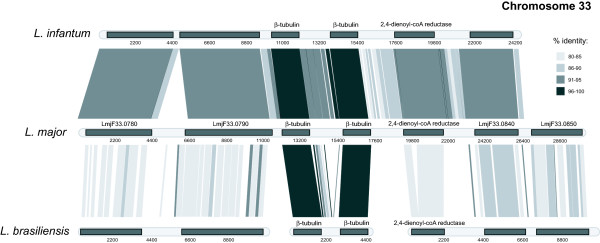
Comparison of the genomic region around the chromosome 33 β-tubulin copy in three *Leishmania *species. For each species, coding regions are represented by shaded rectangles (scale in base pairs) along the chromosome. Contigs were assembled for *L. infantum *and *L. braziliensis *from sequence reads and each assembled contig was aligned with the *L. major *genomic region using the Artemis Comparison Tool (ACT). Blocks of percentage identity exceeding 80% are shown between chromosome cartoons. Locus identifiers refer to the published annotation .

Tiling reads from the *L. infantum *and *L. braziliensis *libraries produced contigs homologous to the chromosome 33 β-tubulin locus (appended as additional files 3 and 6). The karyotype and linkage structure of *L. major *is common also to *L. infantum and L. braziliensis *[18,19], and hence, the chromosomal position of these loci is assumed to be conserved. Figure 1 demonstrates that gene order was conserved both up- and downstream of the β-tubulin array (which is shown with only two β-tubulin copies since the precise number cannot be ascertained from tiling alone). For *L. infantum*, tiling inwards from either flanking gene arrived at β-tubulin and tiling outwards from either the N- and C-terminus of β-tubulin arrived at another copy of the β-tubulin. For *L. braziliensis*, tiling outwards from β-tubulin arrived at another copy, confirming the presence of an array (though see below). It was not possible to tile inwards from flanking genes, although these are present in a conserved gene order, because of sequence gaps either side of the putative array. Despite the lack of any notional links between the β-tubulin array and chromosome 33, there was a physical link between β-tubulin and a 40S ribosomal protein, which is found upstream of the chromosome 33 locus in *L. major*. Likewise, there was a physical link between the *L. infantum *β-tubulin and an ATPase (LmjF33.1010) found on chromosome 33 in *L. major*. Hence, despite the sequence gaps either side of the arrayed genes, these physical links provide contextual evidence for β-tubulin tandem arrays on chromosome 33 in all three species.

**Figure 2:**
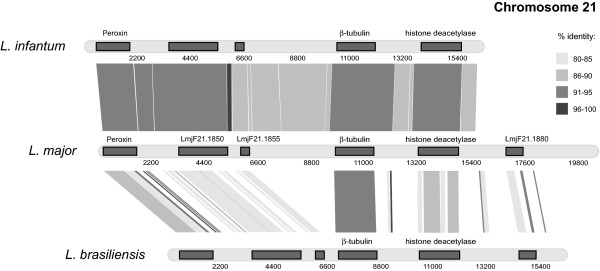
Comparison of the genomic region around the chromosome 21 β-tubulin copy in three *Leishmania *species.

The genes flanking the chromosome 21 singleton β-tubulin in *L. major *were clearly represented in the two other species, as shown in Figure 2. Tiling inwards from these starting points revealed conserved synteny with the chromosome 21 locus and arrived at β-tubulin, although the upstream intergenic sequence (IGS) *in L. braziliensis *is substantially shortened. In *L. infantum *there were physical links between β-tubulin and the upstream IGS (beyond the point of homology with chromosome 33). In *L. braziliensis *there were physical links between β-tubulin and both flanking genes, orthologs to chromosome 21 loci in *L. major*. On this, as on other chromosomes, β-tubulin retained the highest sequence homology between species, other loci being more labile. Generally, the non-coding sequences in *L. braziliensis *were not well conserved (with some exceptions, see below), in contrast to *L. infantum*. These contigs are appended as additional files 2 and 5.

**Figure 3:**
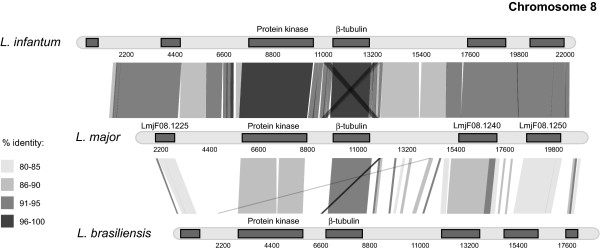
Comparison of the genomic region around the chromosome 8 β-tubulin copy in three *Leishmania *species.

Assembling homologs to the *L. major *chromosome 8 locus was complicated by the presence of a repetitive region, duplicated such that it was on both sides of the β-tubulin gene. When tiling upstream from the gene, the presence of this motif meant that notional links were erroneously inferred with the downstream duplicate (and the same being true in reverse). As a result, using BLAST alone to tile sequence reads gave the impression of tandem β-tubulin genes. While this error has been avoided in the *L. major *genome sequence, it was possible to produce the same effect when tiling *L. major *reads for the corresponding region. However, after accounting for these duplicated, repetitive regions, tiling inwards from flanking genes in both *L. braziliensis *and *L. infantum *produced contigs that were homologous to the *L. major *locus (see Figure 3 and additional files 1 and 4). As with both other loci, there was conserved synteny in both directions and there were physical links with other parts of chromosome 8 to corroborate the notional links implied by read tiling.

### Comparison of flanking regions

All three β-tubulin loci in *L. major *have distinct 3' UTRs. Within the tandem array on chromosome 33 the IGSs between each pair of repeats was conserved at 2256 bp and these had between 98% and 100% sequence identity. Therefore, 3' UTRs within the array were near-identical, with the exception of the last copy; here the 3' UTR was almost entirely different, showing sequence homology with the internal IGS for only 15 bp after the stop codon. Due to sequence gaps, it was not possible to determine if each locus had a distinct 3' UTR in *L. infantum *and *L. braziliensis *by tiling; however, of the three unique 3' UTRs that could be discerned from BLAST searching in these species, two were associated with the singleton loci and therefore, this suggests that the array only comprises a single type of 3' UTR. It is not possible to comment on the final 3' UTR of the *L. braziliensis *tandem array because this data was not available; the final 3' UTR in *L. infantum *tandem array was quite unlike the internal IGSs.

The singleton loci have different UTRs to the tandem array. However, in *L. major *the chromosome 21 and 33 loci shared 225 bp immediately upstream of the start codon and 15 bp immediately downstream of the stop codon. Beyond these regions, their UTRs were unalignable. Neither of the UTRs flanking the chromosome 8 locus in *L. major *shared homology with the other two loci. This situation was replicated in *L. infantum *and *L. braziliensis*, where the chromosome 21 and 33 copies partially shared 5' UTRs, while 3' UTRs were largely unique in all cases.

Comparisons between species reflected their known phylogenetic relationships; the *L. major *and *L. infantum *IGSs were highly conserved (generally in excess of 90% sequence identity), whereas the IGSs in *L. braziliensis *were divergent and seldom exceeded 90% identity. Despite obvious divergence, when the 3' UTRs from the chromosome 33 and 21 assemblies in *L. braziliensis *were BLASTed against all sequences in GenBank, their highest match was with corresponding regions in other *Leishmania *spp. (4e^-12 ^and 4e^-7 ^respectively). The identity of two *L. mexicana *β-tubulin isoforms deposited in GenBank was uncovered by comparing the distinct UTRs of each locus. This showed that one isoform (M23441, [20]) closely matched the chromosome 33 3' UTR (86 bp, 88% identical), while the amastigote-specific isoform (AF345947, [10]) closely matched the chromosome 21 locus (5' UTR: 67 bp, 85% identical; 3' UTR: 750 bp, 84% identical).

Figures 1, 2, 3 clearly show that, although there was a general divergence between *L. major/L.infantum *and *L. braziliensis *IGSs, there were particular motifs in the 3' UTRs of each *L. braziliensis *locus that display a conspicuously high sequence conservation with their homologs in old-world species. Figure 1 identifies two regions of the β-tubulin tandem array IGS in *L. braziliensis*, located 246 bp (5' GTGTACCGCAGTTGTTGGATCCGG 3') and 903 bp (5' GCACTCGCGTTTTCTCGACGGCCCT 3') downstream of the β-tubulin respectively, showing 100% and 96% sequence homology respectively. Similarly, a region 894 bp downstream of the chromosome 21 β-tubulin was 96% conserved between *L. major *and *L. braziliensis *(5' CATGCCTGCCTCTGTAGGTGCTTGTGCGT 3'). Finally, two regions 505 bp (5' ACGCGCGCACTGCGGCACGGAGCGGGAGCTC 3') and 2788 bp (5' TGGCCACTTCCACCGCCACAGCACTCGCC 3') downstream of chromosome 8 locus were 93% identical to corresponding positions in *L. major*.

### Sequence variation among isoforms

**Figure 4:**
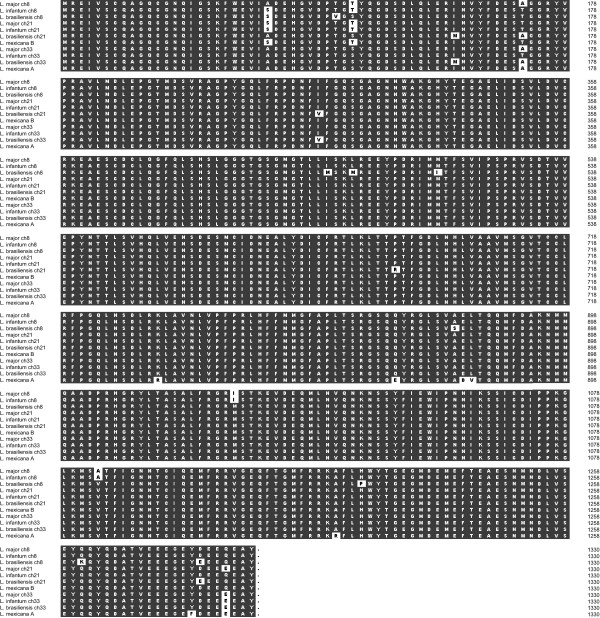
Multiple sequence alignment of β-tubulin isoforms from four *Leishmania *species, ordered by locus. Amino acid substitutions are shown in white.

The two methods of read tiling and physical links produced identical sequences for the six β-tubulin isoforms of *L. infantum *and *L. braziliensis*. The total amount of variation among isoforms from four species was very low (average pairwise divergence: 0.0241), Figure 4 shows that only nine amino acid sites displayed any phylogenetically-informative variation. These substitutions were not diagnostic for either species or locus identity, that is, sequence similarity by species or locus could not be predicted based on amino acid replacements. For example, while the chromosome 21 and 33 assemblies for *L. braziliensis *share unique substitutions (I47M and I91V), these were not shared by the chromosome 8 isoform. Conversely, while chromosome 33 isoforms from all three species share the gln_440 _substitution, this was also found in the *L. major *chromosome 21 isoform. Of all the informative changes, the isoforms appear to vary most at two sites towards the N-terminus (A25S and S35T) and at the C-terminus (E440Q).

### Phylogenetic relationships

**Figure 5:**
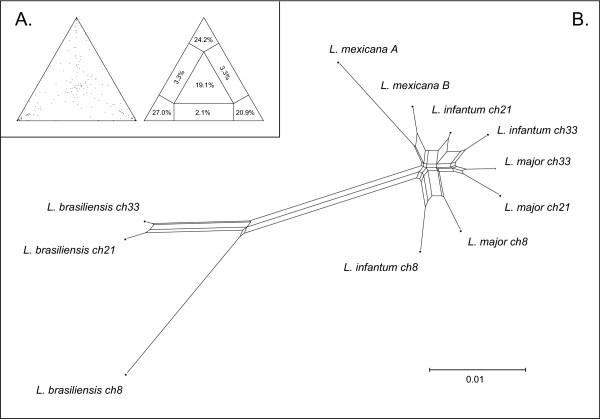
Phylogenetic network of β-tubulin isoforms of *Leishmania *spp. Scale in genetic distance (HKYmodel). (Inset) (A) Likelihood mapping of sequence quartets (left) showing the percentages of resolved, conflicting and unresolved topologies (right).  (B) Phylogenetic network of β-tubulin isoforms of *Leishmania* spp.  Scale in genetic distance (HKY model).

Likelihood mapping of sequence quartets was used to visualise phylogenetic signal among β-tubulin isoforms. The results are shown in Figure 5; these demonstrate that a large proportion of sequence quartets produced unresolved tree topologies (equivalent to the central portion of the triangle: 19.1%). When this is added to those quartets with conflicting resolutions (equivalent to the each side panel), a total of 27.8% of quartets could not be resolved unambiguously. The prevalence of ambiguous relationships was reflected in the ML phylogenies (not shown), which were normally unresolved and not supported by high bootstrap proportions. The exceptions to this pattern were the nodes uniting chromosome 8 isoforms from *L. major *and *L. infantum *(bootstrap value = 76) and the three *L. braziliensis *sequences (bootstrap value = 100). The phylogenetic network shown in Figure 5 shows a consensus of all possible solutions to the phylogenetic tree, emphasising the various, and equally plausible, ways of depicting isoform relationships. No clear definition can be made between chromosome 33 and 21 isoforms in *L. major, L. infantum*, and to some extent, *L. mexicana*, but *L. braziliensis *isoforms are robustly monophyletic.

### Analysis of sequence variation

No evidence was presented by d_N_/d_S _ratios for positive selection; on the contrary, values for ω were consistently less than 1, indicating that β-tubulin sequences have been under purifying selection. Values for ω ranged from 0 (between chromosome 21 isoforms from *L. mexicana *and *L. infantum*) and 0.335 (between *L. mexicana *isoforms). Values for ω when comparing *L. braziliensis *with any other species were significantly lower than when comparing species other than (or within)*L. braziliensis *(t = 3.553, df = 53, p < 0.001). Thus, the number of non-synonymous substitutions was noticeably reduced in genetic distances over the greatest time periods.

There was no evidence for recombination among isoforms; both DSS and PDM methods showed that there were no significant differences in phylogenetic signal along the length of the sequence alignment. Therefore, all nucleotide sites support a single phylogenetic history. However, as Figure 5 shows, there was considerable ambiguity as to the precise branching patterns in this history. Given that this ambiguity largely derived from a lack of information, it follows that the test for recombination is limited because few significantly different topologies will be produced by a data set that lacks signal generally. In any event, the general conservation of these sequences makes site-specific recombination unlikely since this is a diversifying process.

## Discussion

This study has established that all three β-tubulin loci, and the single α-tubulin locus, found in *L. major *are present in *L. infantum *and *L. braziliensis*. Amino acid replacements among β-tubulin sequences are scarce; those that occur are generally idiosyncratic but certain substitutions warrant further analysis. Of particular note are the replacements affecting the C-terminus; at position 440 all array isotypes possess a glutamic acid residue, while all singleton isotypes (with the exception of the *L. major *chromosome 21 copy) have a glutamine. Additionally, the *L. braziliensis *chromosome 21 and 8 copies have an aspartic acid to glutamic acid replacement at position 437. These E440Q and D437E replacements fall within the 'axoneme-specific motif', which is important for flagellar function [21,22], consistent with evolutionary specialisation of these isotypes to life stages with modified flagellar function. Conversely, there are no replacements affecting the region important for post-translational glutamylation [23], located upstream. Towards the N-terminus, the A25S and S35T replacements also seem to preferentially affect the singleton loci and this may be important; it is the amastigote-specific locus that possessed a serine at position 25, as is normal for most eukaryotes, whereas alanine was at this position in all the arrayed paralogs. In a gene where concerted evolution and negative selection are strong, constant pressures for conservatism, it is unlikely that these replacements are the result of neutral divergence. Indeed, there was a consistent under-representation of silent changes. Hence, while most of the evolutionary change associated with these loci has occurred in the non-coding regions, these few coding substitutions may yet prove to be functionally significant.

Comparison of flanking regions supports the view that these loci produce unique transcripts with specific expression profiles. The conservation of gene order around each locus confirms that these three loci are paralogous, i.e., that the ancestor of these three *Leishmania *species possessed a β-tubulin tandem array on chromosome 33 and singleton β-tubulin on chromosomes 21 and 8. In a clear contradiction of these conserved gene orders, the phylogenetic relationships suggested by Figure 5 indicate that paralogs within *L. braziliensis *and *L. mexicana *are more similar to each other than any are to their orthologs in other species. This situation is not extended to isoforms in *L. major *and *L. infantum*, where the precise relationships are unclear.

### UTR structure and the regulation of β-tubulin repertoire

Leishmanial β-tubulin isoforms display a low substitution rate in coding regions but a substantial structural divergence of UTRs. Comparisons between species demonstrate that non-coding sequences rapidly lose structural homology over time, although isolated sections of highly conserved sequence may point to functional constraints on IGS divergence; the first conserved domain described for chromosome 33 above contains a *Leishmania *RNA-binding site GGATC [2]. Besides diverging after speciation, the most important feature of these comparisons is that UTRs are typically dissimilar among loci and within a locus in the case of the tandem array. The exceptions to this are the similarity between the chromosome 33 and 21 loci in the 5' UTRs, and the very beginning of the 3' UTRs. These exceptions notwithstanding, the unique genomic environments around each locus corroborate the three distinct *β-*tubulintranscripts of 2200 bp, 2800 bp and 3200 bp, identified in *L. major *by [2], each predominantly expressed in the promastigiote, amastigote and infective metacyclic life stages respectively. Alignment of the cDNA deposited by [2] with the genome sequences shows that the 2200 bp transcript (GenBank: X93566) corresponds to the internal tandem array copies and the 2800 bp transcript (GenBank: X93567) corresponds to the chromosome 21 singleton. It is now clear that these different transcripts are facilitated by divergence in the 3' UTRs of the various loci, including the terminal UTR of the tandem array, which is quite unlike its internal counterparts, and results in the distinct 3200 bp transcript in the metacyclic stage. Hence, it is likely that these very conservative proteins have acquired diverse expression profiles through restructuring of their flanking regions. This reinforces the view that 3' UTR structure is crucial to the expression profile of any gene through post-transcriptional regulation (rather than promoter regulation) in trypanosomatids [4].

It is intuitive that the various life stages of the *Leishmania *spp. could be the basis for differentiation of β-tubulin isoforms. The promastigote inhabiting the insect gut, the non-proliferating metacyclic form waiting in the insect salivary gland for passage to a vertebrate, and the amastigote, aflagellate within the mammalian macrophage, each experience a different environment and may have suitably different β-tubulin expression requirements. Unique flanking regions described here demonstrate that the amastigote-specific transcripts identified in *L. major *[2] and *L. mexicana *[10] derive from the chromosome 21 locus. The 2200 bp and 3200 bp transcripts identified by [2] originate at the tandem array. The role of the chromosome 8 locus, with its distinctive UTRs, remains elusive; given that it appears more distinct from the two other loci, in both coding and non-coding respects, this gene may fulfil a more distinctive role and could conceivably vary in spatial expression as well as temporal. In summary, the distinctive genomic environments surrounding each β-tubulin locus in *Leishmania *suggest that these different loci satisfy a demand for diverse expression patterns, rather than structural innovation. In the context of poly-cistronic transcription, this could not be delivered by the tandem array on chromosome 33; the conserved flanking sequences within the array offer no opportunities for differential regulation of the copies, except for the terminal UTR, which has been extensively modified. Therefore, paralogous loci may have evolved in response to the derivation of new life stages in *Leishmania*.

### Convergent evolution via ectopic gene conversion

The relationships in Figure 5 show that the phylogeny of β-tubulin genes in *Leishmania *has not simply been a matter of diversification through the inheritance of orthologs. There was insufficient evolutionary change among the isoforms from *L. major *and *L. infantum *for their relationships to be resolved. However, the monophyly of the three *L. braziliensis *isoforms was robustly resolved, which suggests, paradoxically, that they are each other's closest relatives. These loci are clearly paralogous and the conserved gene order across three species demonstrates that all three loci precede the separation of those species from their common ancestor. There was no evidence to support either positive selection towards some consensus sequence or site-specific recombination; in fact, the d_N_/d_S _ratio was always less than 1 and indicative of purifying selection. Therefore, convergence has not occurred according to a fitness benefit associated with conserving a canonical β-tubulin structure. This result can be explained as a consequence of concerted evolution, which has operated to homogenise the different loci in a time-dependent manner.

In the absence of positive selection or recombination between isoforms, ectopic gene conversion may provide a mechanism for homogenisation. Ectopic gene conversion is the unilateral recombination between non-allelic gene copies and has been shown to be a common and important process in gene family evolution [24]. In contrast to allelic gene conversion, an ectopic event occurs between all or part of unlinked loci. They have been shown to occur among amylase and heat-shock proteins in *Drosophila *[25,26]; it is invoked in the origin of novel multi-histocompatibility complex alleles in mammals [27-29], but is most notably documented among vertebrate haemoglobin genes [30,31]. Ectopic gene conversion is also known to affect *Plasmodium falciparum *[32,33], where it is responsible for the generation of sequence variation among *var *genes [33], which are the basis for immune evasion. This process could account for the homogenisation of β-tubulin paralogs, while also accommodating other observations. First, *L. braziliensis *and *L. mexicana *isoforms are not completely homogenised, but the operation of gene conversion as a conservative influence on sequence evolution does not preclude the continued substitution of bases under mutation or selection pressure. The total divergence of two sequences would be a balance of these pressures. Second, *L. major *and *L. infantum *isoforms are very similar but not homogenised; this would be expected if these species only recently diverged and gene conversion is a relatively rare event between paralogous loci in *trans*. It is known that the frequency of conjugation and subsequent conversion events declines with physical distance between loci and their sequence homology [34,35]. Finally, the flanking regions of β-tubulin paralog have clearly not been homogenised. This is not implausible given that gene conversion often affects particular domains, rather than an entire locus. For instance, the exons of ruminant lyzozymes are known to convert, while the introns and flanking regions diverge [36]. In this instance, the non-coding regions may have diverged under positive selection to provide specific expression profiles for each locus; since all three *Leishmania *species display the same patterns of variation in the UTRs (e.g., chromosome 33 and chromosome 21 loci show partial identity in their 5' UTRs but distinct 3' UTRs), it is likely that divergence occurred rapidly in a common ancestor and has been maintained by purifying selection since. Whereas very strong purifying selection on the coding regions ensured that the structural function of β-tubulin was unchanged. While the conservation of coding regions provides a situation susceptible to conversion, the functional divergence of non-coding regions has abolished identity, thereby precluding homogenisation by homologous recombination, which would presumably be maladaptive if it were to occur.

## Conclusion

Since the origin of the distinct β-tubulin loci in *Leishmania*, there has been substantial evolution of the untranscribed regions, in stark contrast to the conservation of coding sequences among all isoforms. The partial remodelling of UTRs had previously been indicated by distinct transcripts belonging to specific life stages. This variation has now been directly related to variation in the genome sequence. In addition to the tandemly-arrayed isoform on chromosome 33, *Leishmania *spp. possess a singleton locus on chromosome 21, which shares partial affinity with the arrayed isoform in its UTRs, and a further singleton on chromosome 8 that shares no affinity with other UTRs and the expression of which remains uncharacterised. These findings indicate that the evolution of distinct UTRs relating to these loci has been coincident with the modification of their expression profiles. However, the conservation of the β-tubulin protein sequence suggests that the structural function has remained unchanged in the different life-stage contexts. In demonstrating the paralogous nature of the three β-tubulin loci, this study has shown that concerted evolution, perhaps through ectopic gene conversion, has operated to maintain uniformity among coding sequences. Therefore, a subtle balance of concert and divergence in different regions of these loci might have allowed β-tubulin repertoire to evolve during the derivation of parasitism in *Leishmania*, while preserving its fidelity in fundamental cell function.

## Methods

### Contig assembly

Contigs were assembled for each locus in *L. infantum *and *L. braziliensis *respectively by tiling together sequence reads selected from read libraries available at GeneDB . For each tiling movement a 100 bp section from the end of a read was used as a probe for BLAST searching. The BLAST search located another read with overlapping sequence; 100% affinity was required between the probe and matching reads to be accepted. Contigs were built by tiling repeatedly in this manner both inwards (i.e., starting with flanking genes and tiling up- or down-stream) or outwards (i.e., beginning with tubulin and tiling up- or downstream). For all of these assemblies, *L. major *coding sequence (either tubulin or flanking genes) was used to initially probe the read libraries in geneDB. In the event of a sequence break (i.e., where no sequence read overlapped with the probe), the process was restarted by probing with the *L. major *sequence (whether coding or non-coding) homologous to the missing data. The resultant gap in the contig could then be bridged by a physical link, that is, by finding a pair of sequence reads positioned on either side of the gap. Tiled reads were assembled into contigs and annotated in Artemis v7. Contigs from all three *Leishmania *species were aligned in ACT v4, which calculated sequence affinity with *L. major *and base composition for the two putative homologous sequences.

### Isoform assembly

Contig assembly provided isoform sequences through the tiling procedure but it was noted that these may be erroneous where, as in the case of tubulin, a lack of uniqueness means that the correct notional link may not be obtained. The three β-tubulin isoforms possess identical central portions and so using the N terminus to tile downstream does not guarantee the appropriate C-terminus and may result in a chimeric assembly. Therefore, isoforms were assembled according to physical, rather than notional, links. Reads matching to β-tubulin were identified in each library; the corresponding read pair was then retrieved from the libraries. Isoforms were assembled by tiling together all reads with physical links to other parts of a given chromosome.

### Phylogenetic reconstruction

A sequence alignment was created from the assembled isoforms plus two *L. mexicana *β-tubulin sequences deposited on GenBank. These sequences were an amastigote-specific isoform (AF345947, [10]) and another isoform retrieved from a whole genome digestion by probing with chicken β-tubulin (M23441, [20]). The phylogenetic information contained in the alignment was visualised using likelihood mapping in TreePuzzle v4.0 ([37], ). This technique selects all quartets of sequences from the data set and calculates the likelihood value of three resolutions for each, using a general-time reversible (GTR) model [38] estimated from the data. Likelihoods are mapped into an equilateral triangle. Quartets mapping into any corner represent successful resolutions and indicate that signal exists. Those mapping to the centre of the triangle represent polytomies due to inadequate signal between the four sequences. Those mapping to the sides of the triangle reflect difficulties in choosing between resolutions because of conflict within the data.

Likelihood mapping showed that phylogenetic signal was relatively low and conflict between character states relatively frequent. A maximum likelihood phylogenetic tree was estimated using a GTR+I+Γ model, parameterised from the data in PAUP*4.0b [39] and non-parametric bootstrapping [40] was applied through 100 heuristic searches. Given the low resolution observed in the phylogenetic tree, a better method for phylogenetic reconstruction was one that explicitly represented the uncertainty in the data set. Therefore, a phylogenetic network was estimated in Splitstree v4.0 ([41], ), using a neighbour-net algorithm [42] and logdet genetic distances [43] to correct for any potential base composition bias.

### Positive selection

The signature of natural selection can be detected in nucleotide sequences through estimation of the ratio of non-synonymous (d_N_) to synonymous (d_S_) substitutions, known as ω[44-46]. Values of 0 and 1 for ω represent ideal states of purifying selection and selective neutrality respectively. Where d_N _exceeds d_S _this is evidence for positive selection [47,48] Positive selection was a possible explanation for the phylogenetic patterns observed and therefore the sequence alignment was scored for d_N _and d_S _in DNAsp v4.10.3 [49], to estimate for ω for each sequence pair.

### Recombination

Another potential explanation for the phylogenetic estimates was site-specific recombination between β-tubulin genes. Potential recombination break-points can be identified from multiple sequence alignments through a variety of methods, most of which detect significant differences in phylogenetic signal between adjacent sites [50]. A sliding window approach was applied using TOPALi v0.27 [51]. Two different procedures were applied to compare adjacent sequence windows and to detect significant departures from a single phylogenetic history within the same alignment. First, difference of sums of squares (DSS, [52]) compared the fit of genetic distance matrices for two adjacent windows to the same tree topology to produce the DSS statistic, the significance of which was determined through parametric bootstrapping in TOPALi. Second, probabilistic divergence measures (PDM, [53]) were used to compare different tree topologies in adjacent sequence windows using a Markov chain Monte-Carlo approach to estimate marginal posterior probabilities for each topology. This method has the advantage of removing any effects due to rate heterogeneity among the sequences. For both DSS and PDM, the window size was fixed at 100 bp and the step size at 10 bp.

## Authors' contributions

APJ carried out comparative genomics analyses, contig assembly and phylogenetic analyses, and also drafted the manuscript. SV carried out preliminary comparative genomics, participated in the design of the study and evaluation of the results. KG conceived of the study, participated in its design and critically examined the manuscript. All authors read and approved the final manuscript.

## Supplementary Material

Additional File 1 *L. braziliensis *contig containing homolog to *L. major *chromosome 8 β-tubulin locus. 19.8 kb contig assembled from *L. braziliensis *sequence reads containing β-tubulin and surrounding genes, homeologous with the chromosome 8 locus in *L. major*. To be viewed in Artemis. Click here for file

Additional File 2 *L. braziliensis *contig containing homolog to *L. major *chromosome 21 β-tubulin locus. 15.3 kb contig assembled from *L. braziliensis *sequence reads containing β-tubulin and surrounding genes, homeologous with the chromosome 21 locus in *L. major*. To be viewed in Artemis. Click here for file

Additional File 3 *L. braziliensis *contig containing homolog to *L. major *chromosome 33 β-tubulin locus. 25 kb contig assembled from *L. braziliensis *sequence reads containing β-tubulin and surrounding genes, homeologous with the chromosome 33 locus in *L. major*. To be viewed in Artemis. Click here for file

Additional File 4 *L. infantum *contig containing homolog to *L. major *chromosome 8 β-tubulin locus. 22 kb contig assembled from *L. infantum *sequence reads containing β-tubulin and surrounding genes, homeologous with the chromosome 8 locus in *L. major*. To be viewed in Artemis. Click here for file

Additional File 5 *L. infantum *contig containing homolog to *L. major *chromosome 21 β-tubulin locus. 16 kb contig assembled from *L. infantum *sequence reads containing β-tubulin and surrounding genes, homeologous with the chromosome 21 locus in *L. major*. To be viewed in Artemis. Click here for file

Additional File 6 *L. infantum *contig containing homolog to *L. major *χηρoμoσoμε 33 β-tubulin locus. 24.4 kb contig assembled from *L. infantum *sequence reads containing β-tubulin and surrounding genes, homeologous with the chromosome 33 locus in *L. major*. To be viewed in Artemis. Click here for file
